# FACTORS RELATED TO THE FRAILTY OF OLDER PEOPLE WITH STROKE IN TEHRAN

**DOI:** 10.1093/geroni/igad104.2794

**Published:** 2023-12-21

**Authors:** Ali Darvishpoor Kakhki, Elham Aghaali

**Affiliations:** Shahid Beheshti University of Medical Sciences, Tehran, Tehran, Iran; Shahid Beheshti University of Medical Sciences, Tehran, Tehran, Iran

## Abstract

Older people are the fastest growing segment of the population particularly in developing countries such as Iran. Older people encounter not only decreasing physical function but also diseases such as stroke which can lead to their frailty. This study was conducted to describe factors related to frailty of older people with stroke in Tehran. This descriptive, correlational study was conducted on a sample of 170 older people with stroke above age 60 in ten educational hospitals in 2020. A socio-demographic questionnaire, the Zarit Scale of Caregiver Burden, and the Frailty Index for Elders were used for data collection. Content validity and Cronbach’s alpha were used for evaluating the validity and reliability of questionnaires. Data were analyzed with SPSS 25. 55.9% of participants were female and 54.1% lived with their spouse; mean age was 77.18 (± 8.57). 92.4% were at risk for severe frailty, and 7.6% were at risk of frailty. The scores of frailty were associated significantly with increasing family caregiver burden and hospital admission rates, not having treatment insurance, and not having a reliable income source. The best predictors of frailty were family caregiver burden, hospital admission rates, and treatment insurance. The study showed that frailty is very common in older people with stroke and is related to a number of factors. Reducing family caregiver burden and admission rates to hospital, and having treatment insurance, will help prevent or reduce frailty in older people with stroke.

